# Media exposure to climate change information and pro-environmental behavior: the role of climate change risk judgment

**DOI:** 10.1186/s40359-024-01771-0

**Published:** 2024-05-11

**Authors:** Ivana Vrselja, Mario Pandžić, Martina Lotar Rihtarić, Maria Ojala

**Affiliations:** 1 Department of Psychology, Catholic University of Croatia University, Ilica 242, Zagreb, 10 000 Croatia; 2 Faculty of Education and Rehabilitation Sciences, University of Zagreb, Zagreb, Croatia; 3 School of Behavioural, Social and Legal Sciences, Örebro University, Örebro, Sweden

**Keywords:** Media, Climate change risk judgment, Worry, Pro-environmental behavior

## Abstract

**Background:**

The aim of this study was to examine the relationships between exposure to climate change information in traditional and modern media, cognitive and emotional aspects of climate change risk judgment, and pro-environmental behavior (PEB).

**Method:**

A cross-sectional online study was conducted on a quota sample of 1,075 participants (51.9% women) aged 18–79 years. Participants self-reported their exposure to climate change-related information in traditional (e.g. television) and modern media (e.g. social networks), cognitive assessment of climate change risk, level of worry about climate change, and the frequency of PEB.

**Results:**

Structural equation modeling showed a good fit for the parallel mediation model, involving cognitive risk judgment and worry as mediators between exposure to climate change information in traditional and modern media and PEB. Exposure to climate change information in traditional media had indirect effect on PEB through heightened worry, but not cognitive risk judgment. In contrast, exposure to climate change information in modern media had no indirect effect on PEB.

**Conclusion:**

Since the link between exposure to climate change information in traditional media and PEB has been shown to be mediated by climate change worry, it is important to enhance the coverage of climate change in traditional media in Croatia, taking care to offer solutions to reduce possible negative impact on people’s well-being.

**Supplementary Information:**

The online version contains supplementary material available at 10.1186/s40359-024-01771-0.

## Introduction

Although the Intergovernmental Panel on Climate Change and the majority of scientific communities have agreed that human activities persistently modify the Earth’s climate, which could have disastrous effects on specific groups and economic sectors [1], the general public does not always perceive the risk of climate change [2].

In academia, risk perception has been approached and defined in many ways [3]. Due to the interchangeable use of various terms related to risk, such as attitudes, beliefs, cognitions and emotions, Dunwoody and Neuwirth [3] recognised the need to clarify the study of risk. The authors [3] suggested replacing the term “risk perception” with “risk judgement” and emphasised the need to distinguish between the cognitive and emotional dimensions of risk judgment. The cognitive dimension is concerned with whether the risk is voluntary or involuntary, whether it can lead to catastrophic events and the extent to which it poses a threat to future generations [3]. The affective dimension of risk assessment refers to the emotional response, such as worry, anxiety or fear, that people feel in relation to hazards [3].

Identifying the factors that influence climate change risk judgment is crucial, as it has been shown to be one of the most important predictors of whether people will engage in pro-environmental behavior [4]. Pro-environmental behaviors can be defined as individual efforts undertaken with the aim of reducing the impact of human activities on the environment [5]. In recent years, the categorisation of pro-environmental behavior has become more diverse and complex as researchers have broadened and shifted their focus and objectives [6]. ​Stern [5], for example, has divided pro-environmental behavior into four dimensions: Environmental activism (e.g. protests, petitions), non-activist behavior in the public sphere (e.g. volunteering for environmental causes, lobbying for environmental policies), private sphere environmentalism(e.g. use and maintenance of environmentally relevant goods, disposal of household waste and environmentally friendly consumption) and other environmentally significant behaviors (e.g. behaviors that influence organisational decisions). While there is a wide range of pro-environmental behaviors, they share a comparable connotation to behaviors in personal (private) and public domains [6]. This categorization of pro-environmental behavior into private and public is utilised in many empirical studies [e.g., 6, 7, 8, 9], and the extent of association between these two forms of pro-environmental behavior and various factors were analysed in several meta-analyses [10, 11].

In this study, the focus is on pro-environmental behavior in the private sphere, which includes everyday actions such as waste avoidance, environmentally conscious consumption and the use of energy-efficient products and services [6–9]. In contrast to public behaviors, which can influence the behavior of many individuals and organisations but whose impact on the environment is primarily indirect through their influence on environmental policy, behaviors in the private sphere have a direct impact on the environment [12].

Studies have shown that private sphere pro-environmental behavior is significantly predicted by a more pronounced cognitive dimension of climate change risk judgement [13, 14] and that people with more pronounced worry and climate anxiety engage in more private pro-environmental behavior [11, 15–17]. In the present study, we capture the cognitive aspect of climate change risk judgment by assessing the risk of climate change having a negative impact on people’s health, safety or wealth. We also capture the emotional aspect of climate change risk judgment through experienced worries about climate change. This is in line with, for instance, pioneer work by Sjöberg [18] who already in the late 1990’s conceptualized the emotional part of risk judgment as worry, which he showed was different from pure cognitive risk judgements [18]. We define worry as a cognitive-emotional concept consisting of brooding about an uncertain future accompanied with anxiety-like affect [19]. Worry has been used as an emotional reaction to climate change, distinct from for example anxiety and concern in many studies [see, for example 15, 20].

These two dimensions of risk judgement are not only associated with pro-environmental behavior, but studies have shown that they are also related to exposure to climate change information in the media [21–23]. The primary rationale behind these findings is that many risks are brought to people’s attention solely through the media, rather than through personal experience [24]. According to the social amplification of risk framework [25–31], when risk information is communicated, the media can either amplify or mitigate the perceived risk. Regardless of the accuracy and particular content of the information, a large flow of information can serve as a risk amplifier [25]. Repeated reports naturally draw the public’s attention to specific risk issues and away from competing sources of attention. According to Mazur [32], “what is said in news stories matters relatively little compared to the amount and saliency of exposure” (p.151). For this reason, in this study we focused on the amount or frequency of exposure to climate change information in the media.

### Present study

Recent research [24, 33, 34] suggest that it is not valid to assume a direct link between exposure to climate change information in the media and pro-environmental behavior. A cross-sectional study by Paek and Hove [24] found that risk perception and negative emotions such as despair, anxiety and fear are mediators between exposure to climate change information in the media and the intention to engage in pro-environmental behavior. Greaves and associates [33] reported that participants showed a significant increase in their negative emotions and intention to engage in pro-environmental behavior after watching a video about climate change compared to those who had not seen the video. Shao and Yu [34] demonstrated that eco- anxiety acts as a mediator between climate change coverage in everyday life and pro-environmental behavior.

To date, there are no studies that simultaneously examine the cognitive dimension of risk judgment and worry as a mediators between exposure to climate change information in different media and pro-environmental behavior. In this study, we distinguish between exposure to climate change information in traditional media (television and radio) and in modern media (social networks and video content sharing channels), which is consistent with the approach of classifying media channels proposed by other researchers [35, 36]. Different approaches are employed in categorising media within research on climate change risk judgment. Some studies [21] analyse media in its most comprehensive form, encompassing all sorts of media, whereas others [22] concentrate on specific media channels. Based on a survey in 110 countries, Thaker [21] reported that exposure to climate change news in the media in general (television, newspapers, social media or conversations with family and friends) is related to the personal cognitive aspect of climate change risk judgment. Another study showed that exposure to climate change information through television predicted higher risk perception in India, but internet use showed a negative effect and newspaper use showed no effect [22]. It could be that these contradictory results are related to the varying degrees of trust in the media examined. Trust in the media conveying risk information is the key factor in whether amplification occurs [37]. Amplification effects are more likely to occur when risk information comes from sources that are highly trusted [37]. Conversely, media sources that are viewed as untrustworthy or as sensationalizing information may have a smaller effect on reinforcing or reducing risk perceptions [37]. These mechanisms contribute to the diffusion of the understanding of a risk, both among individuals directly impacted and within the broader society [31]. In the Croatian context, traditional media such as radio and television are reported to enjoy a higher level of trust than modern media such as social networks [38].

Therefore, this study attempts to answer two key research questions that have not been in focus before. The first research question was whether there is an indirect effect of exposure to climate change information in traditional media on pro-environmental behavior through the cognitive aspect of climate change risk judgment and worry about climate change (R1). The second research question was whether there is an indirect effect of climate change information in modern media on pro-environmental behavior through the cognitive aspect of climate change risk judgment and worry about climate change (R2).

## Method

### Participants

A total of 1075 participants (51.9% women) aged 18 to 79 years participated in this study. Most participants (53.6%) reported a monthly household income of between €1,131 and €2,720. Both high (€2,721 or more) and low income households (€1,130 or less) were less frequently represented in the sample (24.4% and 22% respectively). Furthermore, most participants (66.2%) rated their standard of living as average, with only a handful of them rating their standard of living as far below average (1.2%) or far above average (1.2%).

We employed a quota sampling approach for our research, whereby we selected participants from the adult population of Croatia. The selection was based on specific quotas established according to the geographic location and sex of the participants.

Croatia is administratively divided into 21 counties, each treated as distinct categories for the sampling process. This categorization was done to consider climatic variations within the country, as Croatia is exposed to three different climatic zones [39], and these climatic differences are associated with varying impacts of climate change, such as experiences with extreme weather events [40]. Indeed, previous research has shown that personal experiences with extreme weather events can influence an individual’s engagement with environmental issues [41]. Within each county, the participants were divided by sex to ensure that the proportion of males and females in the sample matched that of the overall population.

The required total sample size was first calculated for a confidence level of 95% and a margin of error of 3%, assuming a population size of 3,204,957 legal adults. The required sample size was estimated at 1,075 participants. To determine the size of each subgroup, a proportional allocation method was adopted. This method entailed allocating a proportionate number of participants based on the respective population sizes within each county and sex category. By following this approach, the intention was to construct a sample that accurately mirrored the distribution of the overall population concerning both geographical location (county) and sex.

To establish the precise number of respondents within each subgroup for both participant’s county and sex criteria, authoritative data from the State Agency for Statistics was utilized. Specifically, information gleaned from the most recent census of the Republic of Croatia, conducted in 2021 by Croatian Bureau of Statistics [42], was employed as the basis for these determinations.

### Instruments

*Exposure to climate change information in the media* was measured using four questions developed specifically for this research. Respondents were asked if they had seen, heard, or read anything about climate change in a list of possible channels: Television, radio, social media (Twitter, Facebook, Twitter, etc.), and video content sharing channels (e.g., YouTube). Respondents indicated their answers on a 5-point scale ranging from 0 (never) to 4 (at least once a day). Based on this list, we specified two-factor CFA measurement model of exposure to climate change in traditional and modern media, where both factors were represented with two indicators (television and radio for traditional, and social networks and video content sharing channels for modern media) and allowed to covary. This model showed good overall fit to the data (*χ2*(1) = 7.417; *p* < .05; CFI = 0.99; TLI = 0.96; RMSEA = 0.08; SRMR = 0.01).

*The cognitive aspect in assessing the risk judgment of climate change (CRJ)* was measured with three questions constructed for this research and based on measurement of climate change risk perception used in Kahan et al. [43]. In original, this measure asks respondents to indicate “How much risk’ they believed ‘climate change’ pose to human health, safety, or prosperity” on a 0 (no risk) to 10 (extreme risk) scale. In this modified version, respondents were asked to give answers to three questions on the level of risk that climate change will pose negative influence to (1) human health, (2) safety and (3) prosperity. Participants indicated their answers on a 11-point scale (0- no risk; 10- extreme risk). To assess construct validity of this measure we specified three-item one-factor measurement model. However, since this model was just-identified and had zero degrees of freedom, the goodness-of-fit of this model could not be analyzed separately and was further explored in the overall measurement model with all latent variables used in the study. SEM-based reliability coefficient for CRJ scale was 0.916, indicating excellent reliability.

*Worry about climate change* was measured with five items taken from Ojala [44]. This measure was applied for the first time to a Croatian sample and back translation was carried out. This measure involved asking respondents about their worries regarding the adverse outcomes stemming from climate change, encompassing concerns for themselves, their loved ones, future generations, people in economically disadvantaged nations, and the animals and nature. Each item was rated on a 5-point scale (1 – not at all; 5 - very much. To assess construct validity of the worry about climate change latent construct, five-item one-factor CFA measurement model was specified. This model showed poor fit to the data (*χ2*(5) = 761.431; *p* < .05; CFI = 0.82; TLI = 0.64; RMSEA = 0.38; SRMR = 0.08). Inspecting modification indices suggestions revealed that specification of residual covariance between first (“I worry about that I myself will be negatively affected by the climate change problem”) and second (“I worry about that my friends and/or my family will be negatively affected by the climate change problem”) indicator would improve the model fit to a great extent. It seems that participants could potentially be equally worried about negative consequence caused by climate change for themselves and their close ones implying that those two variables could share some of their unique variance. In other words, it seems that some of the variance of these two indicators, could be explained with this possibility, besides the proportion explained by underlying common factor. After allowing the covariance between error terms of these two indicators, the model fit improved greatly on most used goodness-of-fit indices (*χ2*(4) = 49.013; *p* < .05; CFI = 0.99; TLI = 0.97; RMSEA = 0.10; SRMR = 0.02), where only RMSEA index was over the suggested 0.06 threshold. Also, all the standardized factor loadings were high, indicating that each of the five indicators had high saturations with the latent construct. SEM-based reliability coefficient of this scale was 0.865, implying very good reliability.

*Pro-environmental behavior* was measured with 8 items taken from Ojala [44, 45]. This measure was applied for the first time to a Croatian sample and back translation was carried out. The items contained both everyday behavior (e.g., “cycling or walking instead of being driven by car”) and communicating the need to do something about the environment to other people (e.g., “trying to influence one’s friends or/and peers to care more for the environment”) (see Supplementary Material 1 for descriptive statistics and intercorrelations among pro-environmental behavior scale items). Each item was assessed on a 5-point scale (1 - almost never; 5 - almost always). To assess construct validity of this scale, eight-item one-factor measurement CFA model was specified. Goodness-of-fit indices suggested poor fit of the model (*χ2*(20) = 341.890; *p* < .05; CFI = 0.81; TLI = 0.73; RMSEA = 0.12; SRMR = 0.07). Most indicators were poorly saturated with this latent construct, where standardized factor loadings were under or just around 0.50. Hence, only the three indicators with highest factor loadings were kept (i.e., *Try to influence my family and friends to act in a climate-friendly way*; *Save energy in the household*; *Make climate-friendly food choices*), and a new three-item one-factor measurement model was inspected. The goodness-of-fit of this model, however, could not be analyzed since the model had zero degrees of freedom and was just-identified, and it was further explored in the overall measurement model with all used latent variables. After removing five indicators the SEM-based reliability coefficient slightly dropped, from 0.745 to 0.705, indicating that reliability of this shorter measure was still good.

### Procedure

The study was part of a larger research project entitled Sociopsychological Determinants of Climate Change Risk Perception and Possibilities for its Amplification, funded by the Catholic University of Croatia.

This study was approved by the Ethics Committee of the Catholic University of Croatia and was conducted online between March and June 2023. The survey instrument was created using the SoSci Survey application [46] and was accessible to participants through the website www.soscisurvey.de. Study participants were recruited with the active participation of the research team members, who used social media platforms (Facebook, Twitter, Instagram, LinkedIn, etc.) to distribute recruitment flyers with the link on the research. In addition, the researchers sent these recruitment flyers via communication applications (e.g. Whatsapp, Viber, etc.) to share the invitation with their friends and colleagues. After accessing the link to the study, participants were informed about the aims of the study, the procedures and their rights as participants before completing the questionnaire. They were assured that their answers would remain anonymous and that no data would be collected during the research that could potentially reveal their identity. Participants were informed that their data would only be aggregated at group level and would only be used for research purposes. It was explicitly stated that participants could withdraw from the study at any time and contact the researchers if they had any concerns or questions. After reading this section, participants were asked to give their consent to participate in the study by clicking the ‘Continue’ button. Those who agreed to participate in the study then completed the questionnaire. The questionnaire took approximately 20 min to complete.

### Data analysis

To explore the research questions, structural equation modeling (SEM) was implemented. All analyses for hypothesis testing were conducted using the *lavaan* package [47] in R [48], while figures were produced using the *semPlot* [49] and *semptools* [50] packages. Model fit assessments were not solely based on chi-square statistic given its high sensitivity to sample size [51]. Hence, other goodness-of-fit indices (CLI, TLI, RMSEA and SRMR) based on this statistic were inspected following cut-off values guidelines proposed by Hu and Bentler [52]. Reliability coefficient of the used scales was SEM-based, and it was calculated as a ratio of explained and total variance of the latent variable indicators. (for more information on composite reliability see [53]).

## Results

Table 1 shows the distribution of participants according to how often they received information about climate change through traditional or modern media.

**Table 1:** Distribution of Participants by frequency of exposure to climate change information in different media

Type of media		Frequency of exposure (%)				
		Never	Several times per year	Several times per month	Several times per week	At least once a day
Traditional	Television	8.7	23.3	43.1	19.7	5.1
	Radio	27.4	29.7	27.9	12.3	2.7
Modern	Social media	12.3	13.5	29.5	30.5	14.3
	Video platform	22.7	21.2	31.9	18.1	6.2

When traditional media is considered, television exposure to climate change information, in comparison to radio exposure, seems to be higher, since almost one quarter of participants (24.8%) indicated that they received climate change information through this media several times per week or at least once a day in comparison to only 15% of the radio listeners. Furthermore, almost 30% of the participants indicated that they were never exposed to climate change information through radio. When we consider modern media channels, exposure to climate change information was more frequent through social media in comparison to video platforms. About 45% of the participants indicated that they were exposed to such information several times per week or at least once a day using social media platforms, in comparison to only 24.3% of them when video platforms are considered. Taken overall, participants were most likely to receive information about climate change through social media and least likely to receive it through radio.

The descriptive statistics for the other variables included in the study is presented in Table 2.

**Table 2:** Descriptive statistics for cognitive aspect of climate change risk judgment, worry and pro-environmental behavior

Variable	M	SD	Possible range
Cognitive aspect	7.90	2.238	0–10
Worry	3.77	0.890	1–5
Pro-environmental behavior	3.13	0.900	1–5

On average, participants judged the risk of climate change to be fairly high. Furthermore, mean level of worry about climate change was above the midpoint of the scale range, while mean value of pro-environmental behaviors was in the middle of the scale range.

Table 3 contains the intercorrelations between the averaged constructs of the study. As can be seen, all correlation coefficients were statistically significant and positive. The relationship between two types of media exposure to climate change information was moderate in strength, while the strength of the relationship between cognitive aspects of climate change risk judgment and worry about climate change was moderate to high.

**Table 3:** Intercorrelations between study constructs

	1	2	3	4	5
1. Traditional media	/	0.407^**^	0.087^**^	0.186^**^	0.259^**^
2. Modern media		/	0.074^*^	0.130^**^	0.169^**^
3. Cognitive aspects			/	0.635^**^	0.339^**^
4. Worry				/	0.393^**^
5. Pro-environmental behavior					/

Note. * *p* < .05, ** *p* < .01

Exposure to climate change information in both types of media channels had a weak correlation with both cognitive aspects of climate change risk judgment and worry about climate change, with relationship between exposure to climate change information in traditional media and worry about climate change was slightly stronger than the rest. Furthermore, pro-environmental behavior had a moderate correlation with both cognitive aspects of climate change risk judgment and worry about climate change. Also, it had low correlation with exposure to climate change information in modern media, and low to moderate correlation with exposure to climate change information in traditional media.

Before assessing the responses to our research questions, overall measurement model (see Supplementary Material 2) that incorporated all constructs in the study and that allowed for inter-latent covariances was specified. This model showed good fit to the data (*χ2*(79) = 273.471; *p* < .05; CFI = 0.98; TLI = 0.97; RMSEA = 0.05; SRMR = 0.04). There was significant positive relationship between most study constructs. In the case of traditional media, exposure to climate change information showed a significant positive relationship with both worry about climate change and cognitive aspect of climate change risk judgment. In the case of modern media, on the other hand, exposure to climate change information showed to be non-related to cognitive aspect of climate change risk judgment, while the relationship with worry about climate change was positive. Finally, there was a significant positive relationship between exposure to climate change information in traditional and modern media, as well as between cognitive aspect of climate change risk judgment and worry about climate change, while all the aforementioned constructs were also positively related to pro-environmental behavior.

**Fig. 1:**
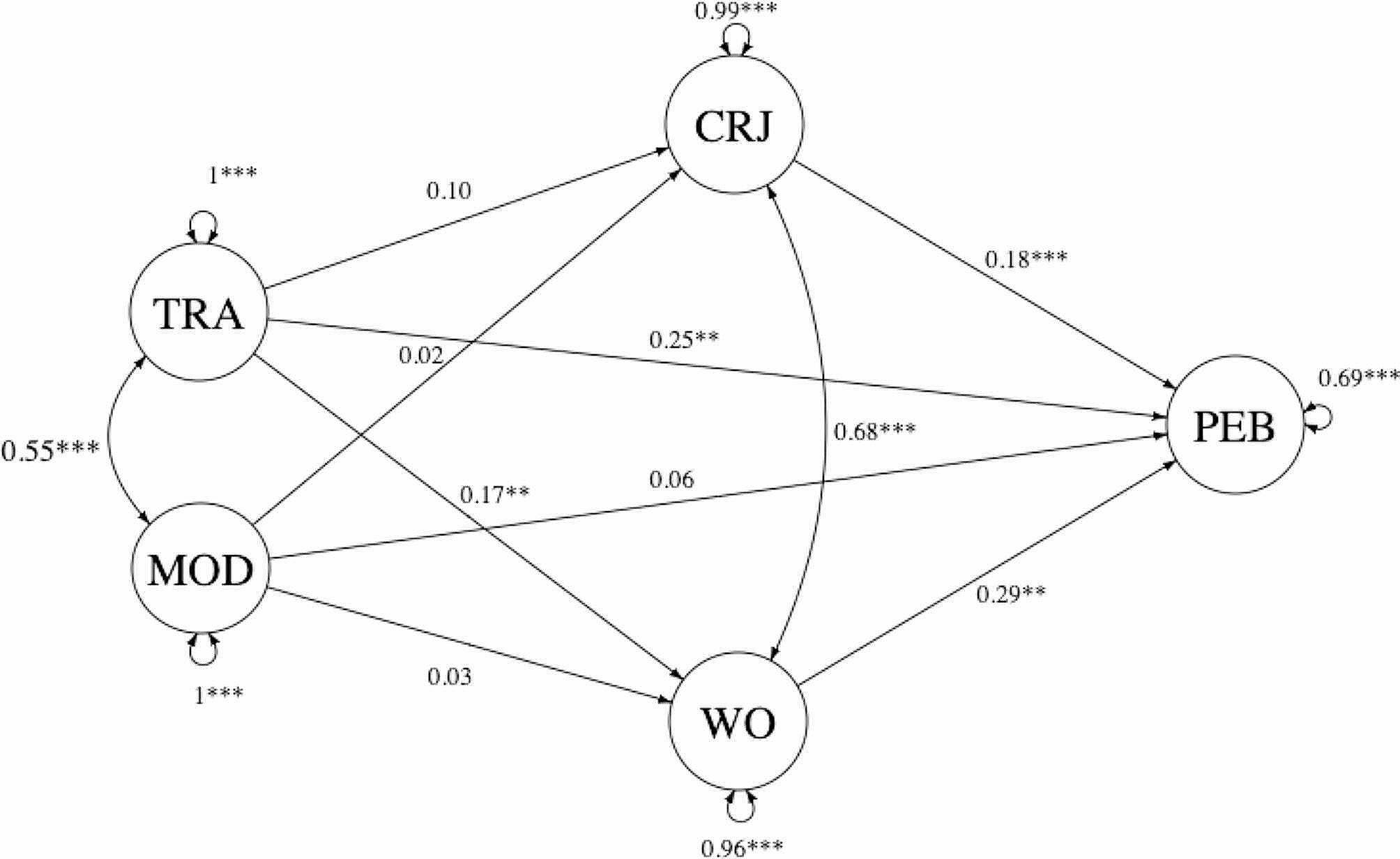
Parameter estimates for the full parallel mediation model. Note.^*^p < .05, ^**^p < .01, ^***^p < .001. Standardized coefficients are presented. Measurement part of the model is omitted. TRA – exposure to climate change information in traditional media, MOD – exposure to climate change information in modern media, CRJ – cognitive aspect of climate change risk judgment, WO – worry about climate change, PEB – pro-environmental behavior

Next, we defined a full structural equation model specifying eight directional paths, and two covariances (between two predictors and between two mediators) among latent variables (Fig. 1).

This parallel mediation model showed good fit (*χ2*(79) = 273.471; *p* < .05; CFI = 0.98; TLI = 0.97; RMSEA = 0.05; SRMR = 0.04). Because there are different approaches to measuring pro-environmental behavior [8, 9], we conducted additional analyzes using individual items underlying the latent construct of pro-environmental behavior as outcome variables (see Supplementary Material 3, 4, 5, and 6 for more details). The results of these analyzes are consistent with the results of the analysis with pro-environmental behavior as a latent variable.

Finally, we tested whether there are indirect effects of exposure to climate change information in traditional and modern media on pro-environmental behaviors through the cognitive aspect of climate change risk judgment and worry about climate change (Table 4).

**Table 4:** Indirect effects of exposure to climate change information in different media on pro-environmental behavior

Type of media	Mediator	*b*	*SE*	*z*	*p*	95% confidence interval	
						lower	upper
Traditional	Cognitive aspect	0.02	0.02	1.61	0.108	-0.002	0.056
	Worry	0.07	0.03	2.66	0.008	0.022	0.119
Modern	Cognitive aspect	0.00	0.01	0.42	0.676	-0.016	0.028
	Worry	0.01	0.02	0.53	0.595	-0.025	0.042

Note. Bootstrap confidence intervals based on 5000 samples are presented

In the case of traditional media, it was shown that there is an indirect effect of exposure to climate change information on pro-environmental behavior, through worry about climate change, but not through the cognitive aspect of climate change risk judgment. Specifically, higher exposure to climate change information in traditional media was found to be associated with greater worry about climate change, which in turn was associated with more frequent pro-environmental behaviors (Table 4). These results suggest a partial positive answer to our research question R1.

In relation to research question R2, the results show that there is no indirect effect of exposure to climate change information in modern media on pro-environmental behavior, neither through the cognitive aspect of climate change risk judgment nor worry about climate change (Table 4).

## Discussion

This cross-sectional analysis of a relatively large sample of Croatian adults shows that exposure to information about climate change in traditional media was associated with people’s increased worry about climate change. Next, this increased worry about climate change was found to be positively associated with people’s pro-environmental behaviors. Interestingly, however, the study found no evidence that exposure to climate change information in traditional media is indirectly associated with pro-environmental behavior through the cognitive aspect of climate change risk judgment.

We found a significant relationship between the cognitive aspect of climate change risk judgment and pro-environmental behavior in our sample. However, exposure to climate change information in traditional media was not significantly associated with the cognitive aspect of risk judgment. One plausible explanation for these results is that television broadcasters are a major source of information for Croatians [54, 55], and these broadcasters are controlled by political actors [56] who have not prioritized climate change [57, 58]. Our results also suggest that only a quarter of our participants regularly received climate change information via TV programs, while the exposure rate via social media channels was almost double that of TV programs. The resulting scarcity of information about climate change in television and other traditional media may create uncertainty in the public [59], which in turn may induce worry about the future [60].

While worrying about climate change might motivate pro-environmental behaviors, as demonstrated in the present work and previous studies, such worry can reduce well-being. Climate anxiety has been linked to depressive symptoms as well as reduced mental health and psychological well-being [61–65]. To counteract these negative effects, media can shift the focus of climate change information away from potential impacts to potential solutions [17]. Future research should systematically examine where the focus of climate change information lies in Croatia and other countries in order to ensure that emerging climate journalism leads to behavioral change without adverse psychological consequences.

The lack of a significant relationship between exposure to climate change information in modern media and pro-environmental behavior in our sample likely reflects Croats’ lack of trust in social media and online news, due in part to concerns about misinformation and disinformation [38]. This mistrust is understandable in light of the sensationalist tone with which topics in climate change have recently been covered in online news portals in the country [66]. That analysis of climate change information in media before and after the UN Climate Summit in 2019 showed that much of the information centered around Greta Thunberg and the climate strikes with which she was associated, rather than on the Summit itself [66]. Many articles focused on the personality of Thunberg and the conflictual aspect of climate strikes without delving into the underlying issues of climate change. Media outlets adopting an active, sometimes alarmist stance on climate change were more likely to focus on positive representations of Thunberg, while those treating climate change with skepticism or outright denial were more likely to focus on negative representations.

Some limitations of this study need to be mentioned. First, our analysis was based on cross-sectional online survey data, and the conclusions about causality cannot be drawn. Further studies that could establish causal relationships between variables are warranted. Second, our measurement of exposure to climate change information in traditional and modern media may be problematic, since the use of retrospective recall to gauge respondents’ average exposure to information about climatic change on media may introduce biases and inaccuracies. Future research should employ real-time measurements and other more accurate assessment methods of media usage.

Third, although the measure of pro-environmental behavior used in this study is widely accepted and has been used in numerous studies outside Croatia [17, 61], our results show that certain indicators within this measure are poorly aligned with the underlying latent construct of pro-environmental behavior. Studies measure pro-environmental behavior in different ways. In this study, we measured different types of pro-environmental behavior that can be grouped under the umbrella term of pro-environmental behavior in the private sphere [5–9]. However, our results suggest that only three of the eight indicators of the pro-environmental measure used are satisfactorily related to the underlying latent construct of pro-environmental behavior. Seemingly different behaviors, such as trying to persuade family and friends to behave in a climate-friendly way, saving energy in the household and eating a climate-conscious diet, form a single behavioral construct. At this point, it is also important to note that we conducted an additional structural equation analysis with these three specific pro-environmental behaviors as outcomes. The results of these analyzes were consistent with those of the analysis using the latent construct of pro-environmental behaviors as outcome. However, as some studies [67] have shown discrepancies in the relationships between the variables studied and different types of pro-environmental behavior, it is crucial for future research to determine whether the relationships observed in this study can be replicated in other types of pro-environmental behavior.

Nevertheless, this study offers some notable advantages. In our online research, we used a quota sampling strategy in which we set specific quotas based on the geographic location and gender of the participants. We chose this sampling method to circumvent some of the common limitations associated with online research, particularly the well-documented tendency for male participants to have lower response rates compared to female participants in previous studies [68]. In addition, this study stands out in the literature because it examines two dimensions of risk judgment-emotional and cognitive-a relatively rare approach. It also makes a valuable contribution to our understanding of how different media relate to risk judgment and pro-environmental behavior.

This study also has practical implications. In Croatia and in other countries where traditional media is more trusted than modern media, it is advisable to use traditional media channels for climate change communication. It is, however, crucial that such communication not only highlights the negative impacts of climate change, but also emphasizes possible solutions to prevent worry about climate change from negatively affecting people’s well-being.

### Electronic supplementary material

Below is the link to the electronic supplementary material.

Supplementary Material 1

Supplementary Material 2

Supplementary Material 3

Supplementary Material 4

Supplementary Material 5

Supplementary Material 6

## Data Availability

The datasets analyzed in the current study are available from the corresponding author on reasonable request.
