# Supplementary material for: Media exposure to climate change information and pro-environmental behavior: the role of climate change risk judgment
Source: BMC Psychol. 2024 May 11;12:262. doi: 10.1186/s40359-024-01771-0 (PMC11088128; doi:10.1186/s40359-024-01771-0)
Supplement: Supplementary file 1 — Supplementary Material 1 [file 40359_2024_1771_MOESM1_ESM.docx]

SUPPLEMENTARY MATERIAL 1

*Descriptive Statistics and Intercorrelations Among Pro-Environmental Behavior Scale Items*

|  | 1 | 2 | 3 | 4 | 5 | 6 | 7 | 8 |
| --- | --- | --- | --- | --- | --- | --- | --- | --- |
| 1. Cycle or walk instead of driving or being  driven in a car (e1) |  | .323^**^ | .248^**^ | .262^**^ | .278^**^ | .512^**^ | .197^**^ | .257^**^ |
| 2. Restrain myself from buying new clothes  that I don’t need (e2) |  |  | .272^**^ | .252^**^ | .357^**^ | .220^**^ | .281^**^ | .224^**^ |
| 3. Choose not to fly (e3) |  |  |  | .255^**^ | .297^**^ | .210^**^ | .206^**^ | .149^**^ |
| 4. Try to influence my family and friends  to act in a climate-friendly way (e4) |  |  |  |  | .490^**^ | .150^**^ | .220^**^ | .460^**^ |
| 5. Save energy in the household (e5) |  |  |  |  |  | .217^**^ | .403^**^ | .343^**^ |
| 6. Take public transportation instead of the car (e6) |  |  |  |  |  |  | .245^**^ | .197^**^ |
| 7. Avoid food waste (e7) |  |  |  |  |  |  |  | .271^**^ |
| 8. Make climate-friendly food choices (e8) |  |  |  |  |  |  |  |  |
| *M* | 3.03 | 3.42 | 3.52 | 3.05 | 3.67 | 2.85 | 4.12 | 2.66 |
| *SD* | 1.15 | 1.15 | 1.46 | 1.17 | 1.03 | 1.45 | 0.93 | 1.22 |
| *Note*. * p < .05, ** p < .01 | | | | | |  |  |  |
