# Supplementary material for: Media exposure to climate change information and pro-environmental behavior: the role of climate change risk judgment
Source: BMC Psychol. 2024 May 11;12:262. doi: 10.1186/s40359-024-01771-0 (PMC11088128; doi:10.1186/s40359-024-01771-0)
Supplement: Supplementary file 2 — Supplementary Material 2 [file 40359_2024_1771_MOESM2_ESM.docx]

SUPPLEMENTARY MATERIAL 2

*Overall Measurement Model Containing all Latent Constructs*


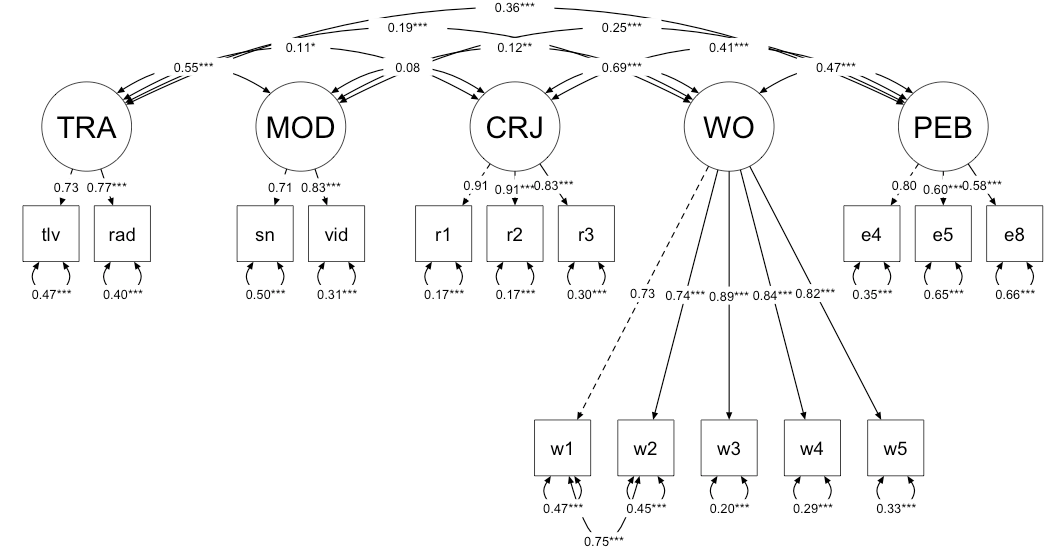


*Note.* ^*^ p < .05, ^**^ p < .01, ^***^ p < .001. Standardized coefficients are presented. TRA – exposure to climate change information in traditional media, MOD – exposure to climate change information in modern media, CRJ – cognitive aspect of climate change risk judgment, WO – worry about climate change, PEB – pro-environmental behavior.
