# Supplementary material for: Media exposure to climate change information and pro-environmental behavior: the role of climate change risk judgment
Source: BMC Psychol. 2024 May 11;12:262. doi: 10.1186/s40359-024-01771-0 (PMC11088128; doi:10.1186/s40359-024-01771-0)
Supplement: Supplementary file 3 — Supplementary Material 3 [file 40359_2024_1771_MOESM3_ESM.docx]

SUPPLEMENTARY MATERIAL 3

*Intercorrelations Between Pro-Environmental Behavior Scale Items and Overall Results on Media Exposure to Climate Change, Worry About Climate Change, and Cognitive Aspect of Climate Change Risk Judgment*

|  | 1 | 2 | 3 | 4 | 5 | 6 | 7 |
| --- | --- | --- | --- | --- | --- | --- | --- |
| 1. Traditional media |  | .407^**^ | .087^**^ | .186^**^ | .249^**^ | .132^**^ | .222^**^ |
| 2. Modern media |  |  | .074^*^ | .130^**^ | .188^**^ | .081^**^ | .128^**^ |
| 3. Cognitive aspects |  |  |  | .635^**^ | .299^**^ | .260^**^ | .242^**^ |
| 4. Worry |  |  |  |  | .373^**^ | .273^**^ | .283^**^ |
| 5. e4 |  |  |  |  |  | .490^**^ | .460^**^ |
| 6. e5 |  |  |  |  |  |  | .343^**^ |
| 7. e8 |  |  |  |  |  |  |  |
| *Note*. * p < .05, ** p < .01 | | | | | |  |  |
