# Supplementary material for: Media exposure to climate change information and pro-environmental behavior: the role of climate change risk judgment
Source: BMC Psychol. 2024 May 11;12:262. doi: 10.1186/s40359-024-01771-0 (PMC11088128; doi:10.1186/s40359-024-01771-0)
Supplement: Supplementary file 4 — Supplementary Material 4 [file 40359_2024_1771_MOESM4_ESM.docx]

SUPPLEMENTARY MATERIAL 4

*Parameter Estimates for the Full Parallel Mediation Model with E4 Item of Pro-Environmental Behavior Scale as an Outcome*

*
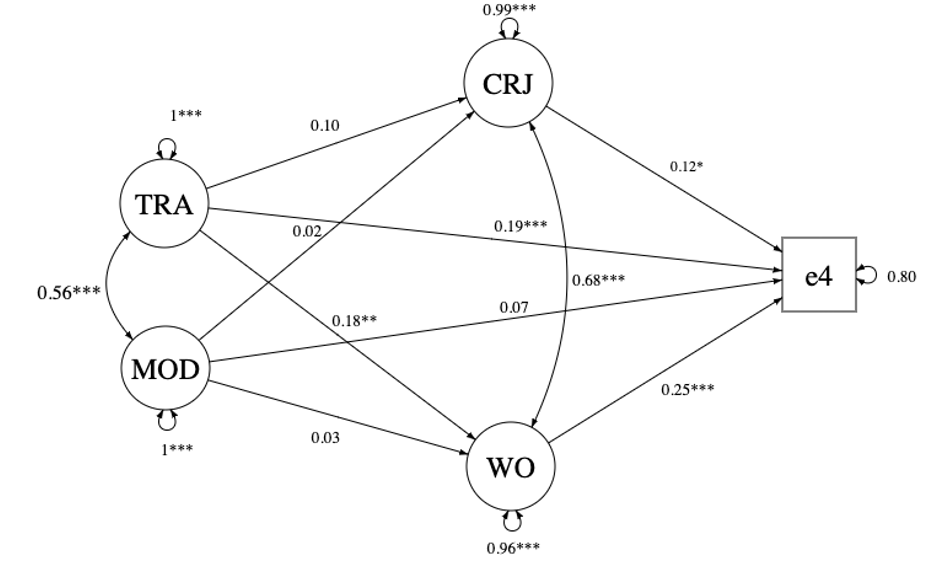
*

*Note.* ^*^ *p* < .05, ^**^ *p* < .01, ^***^ *p* < .001. Standardized coefficients are presented. Measurement part of the model is omitted. TRA – exposure to climate change information in traditional media, MOD – exposure to climate change information in modern media, CRJ – cognitive aspect of climate change risk judgment, WO – worry about climate change. *Χ²* (55) = 213.395, *p* < .001; CFI = 0.981, TLI = 0.973, RMSEA = 0.053, SRMR = 0.035

*Indirect Effects of Exposure to Climate Change Information in Different Media on Item 4 (e4) of Pro-environmental Behavior Scale*

| Type of media | Mediator | *b* | *SE* | *z* | *p* | 95% confidence interval | |
| --- | --- | --- | --- | --- | --- | --- | --- |
|  |  |  |  |  |  | lower | upper |
| Traditional | Cognitive aspect | 0.02 | 0.01 | 1.42 | 0.155 | -0.003 | 0.049 |
|  | Worry | 0.07 | 0.03 | 2.63 | 0.009 | 0.023 | 0.129 |
| Modern | Cognitive aspect | 0.00 | 0.01 | 0.43 | 0.665 | -0.013 | 0.025 |
|  | Worry | 0.01 | 0.02 | 0.52 | 0.604 | -0.028 | 0.046 |
| *Note*. Bootstrap confidence intervals based on 5000 samples are presented. | | | | | | | |
