# Supplementary material for: Media exposure to climate change information and pro-environmental behavior: the role of climate change risk judgment
Source: BMC Psychol. 2024 May 11;12:262. doi: 10.1186/s40359-024-01771-0 (PMC11088128; doi:10.1186/s40359-024-01771-0)
Supplement: Supplementary file 5 — Supplementary Material 5 [file 40359_2024_1771_MOESM5_ESM.docx]

SUPPLEMENTARY MATERIAL 5

*Parameter Estimates for the Full Parallel Mediation Model with e5 Item of Pro-Environmental Behavior Scale as an Outcome*

*
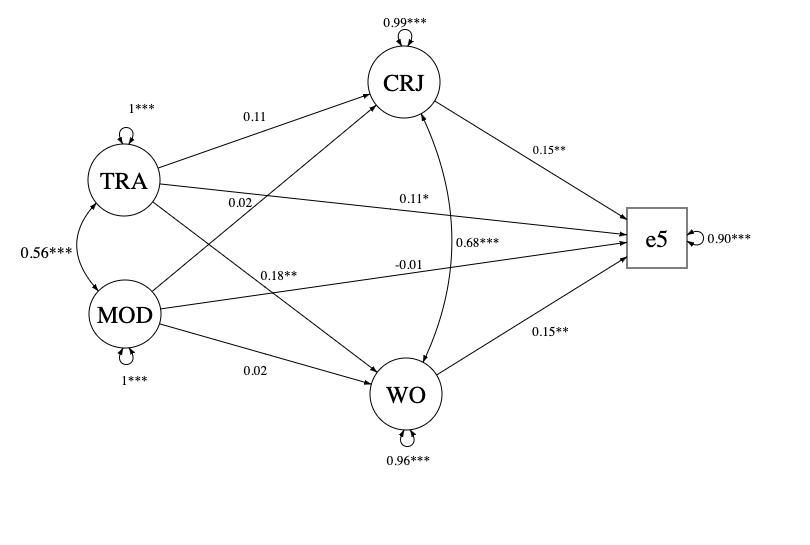
*

*Note.* ^*^ *p* < .05, ^**^ *p* < .01, ^***^ *p* < .001. Standardized coefficients are presented. Measurement part of the model is omitted. TRA – exposure to climate change information in traditional media, MOD – exposure to climate change information in modern media, CRJ – cognitive aspect of climate change risk judgment, WO – worry about climate change. *Χ²* (55) = 196.955, *p* < .001, CFI = 0.983, TLI = 0.975, RMSEA = 0.05, SRMR = 0.033

*Indirect Effects of Exposure to Climate Change Information in Different Media on Item 5 (e5) of Pro-environmental Behavior Scale*

| Type of media | Mediator | *b* | *SE* | *z* | *p* | 95% confidence interval | |
| --- | --- | --- | --- | --- | --- | --- | --- |
|  |  |  |  |  |  | lower | upper |
| Traditional | Cognitive aspect | 0.02 | 0.01 | 1.56 | 0.12 | -0.001 | 0.054 |
|  | Worry | 0.04 | 0.02 | 2.12 | 0.034 | 0.008 | 0.078 |
| Modern | Cognitive aspect | 0.00 | 0.01 | 0.35 | 0.724 | -0.016 | 0.023 |
|  | Worry | 0.00 | 0.01 | 0.43 | 0.668 | -0.015 | 0.026 |
| *Note*. Bootstrap confidence intervals based on 5000 samples are presented. | | | | | | | |
